# Integrated Youth Service Preferences of Caregivers of Justice-Involved Youth: A Discrete Choice Conjoint Experiment

**DOI:** 10.5334/ijic.7044

**Published:** 2024-01-29

**Authors:** Deanna F. Klymkiw, David M. Day, J. L. Henderson, Lisa D. Hawke

**Affiliations:** 1 Department of Psychology, Toronto Metropolitan University, 350 Victoria Street, Toronto, Ontario, Canada; 2 Centre for Addiction and Mental Health, 1000 Queen Street West, Toronto, Ontario, Canada; 3 Department of Psychiatry, University of Toronto, 250 College Street, Toronto, Ontario, Canada

**Keywords:** integrated care, justice-involved youth, caregiver, parent preferences, mental health, substance use

## Abstract

**Introduction:**

Mental health and/or substance use (MHS) challenges affect approximately 95% of youth in the criminal justice system, with only three in ten justice-involved youth receiving treatment. Caregivers of justice-involved youth have identified fragmented care as a barrier to youth accessing MHS services. One suggested solution to this problem is the implementation of integrated youth services (IYS). However, it is unknown which IYS components caregivers of justice-involved youth prioritize.

**Methods:**

Using a discrete choice conjoint experiment (DCE), *n* = 46 caregivers of justice-involved youth, and *n* = 204 caregivers of non-justice-involved, completed thirteen choice tasks representing different combinations of IYS.

**Results:**

Both caregiver groups exhibited preferences for involvement and access to information regarding their youth’s treatment, and fast access to broad range of core health and additional services, in a community setting, with the incorporation of e-health services. Caregivers of justice-involved youth showed a unique preference for involvement in family counseling with their youth. The incorporation of this service feature may help to engage caregivers of justice-involved youth in their youths’ MHS treatment 3-fold.

**Conclusion:**

Data gleaned from this analysis provides an understanding of what components of IYS models may help to engage caregivers of justice-involved youth.

## Introduction

Mental health and substance use (MHS) challenges affect approximately 95% of justice-involved youth internationally, a group overrepresented by Black, Indigenous and People of Color (BIPOC) with low socioeconomic status [1,2]. Although effective early intervention optimizes positive youth outcomes, a minority of youth involved in the criminal justice system receive needed treatment [3]. Unmet MHS challenges in justice-involved youth is problematic, as MHS challenges, particularly substance abuse, increases the risk for recidivism [4,5]. Furthermore, research has demonstrated that justice-involved youth awaiting MHS treatment are at an increased risk for early recidivism compared to justice-involved youth who have completed MHS treatment [6].

Caregivers of youth with MHS challenges have identified fragmented care as a leading barrier to accessing MHS services for their youth [7]. One suggested solution to the problem of fragmented care is the implementation of integrated youth services (IYS), where youth and their caregivers can access evidence-based MHS treatment within a single, community-based location [8]. In recognition of the benefits of the IYS model, pan-Canadian initiatives to implement IYS are occurring in tandem with an international movement toward integrated models of youth MHS service delivery [8]. It is well established that a multisystem approach that addresses the biopsychosocial needs of justice-involved youth is an optimal strategy in supporting this population [9,10,11]. Given that addressing an array of youth needs is paramount to the IYS model [12], IYS are a well-suited solution to delivering care to this underserved population. Indeed, research suggests that an integrated model of MHS care may increase service utilization and treatment completion, and may even lower rates of recidivism, among justice-involved youth [13,14].

While it is recognized that integrated services are needed to solve the problem of fragmented care, it is unknown what components justice-involved youth and their caregivers want to receive within an IYS. Although previous research has begun to explore the IYS service preferences of justice-involved youth [see Klymkiw et al., 2024, in press], the service preferences of caregivers of justice-involved youth remain unknown. This is an important question to consider, given the integral role caregivers can play in their youths’ successful initiation and continued MHS treatment [9,10,11].

### Using Discrete Choice Conjoint Methodology to Understand Service Preferences

Caregivers of justice-involved youth have expressed skepticism in MHS services [15]. However, empowering caregivers to play an active role in MHS service design may enhance their confidence in the services they use [16]. Use of a discrete choice conjoint experiment (DCE) can help to achieve this. In completing a DCE, participants are required to make decisions regarding their preferences among competing hypothetical service options [17]. In doing so, participant choices mimic real-world decision making in that it prompts them to consider what services they prioritize, at the expense of losing other services [18].

Data gathered from an Ontario-wide DCE has examined the IYS service preferences of youth, caregivers, and stakeholder groups [19,20,21]. Most relevant to the current data analysis are findings from the Hawke and colleagues [20] study examining the IYS service preferences of Ontario caregivers that found three caregiver latent classes. The *Comprehensive, Integrative Service Access* class favoured expeditious access to an array of services; the *Service Process Features* class most preferred process features (e.g., age range of services and time of appointments), and the *Caregiver Involvement* class’s strongest preference was for involvement in their youths’ services. Many similar preferences among caregivers were also found; all showed a preference for expeditious access to an array of services, including health services, and the option to be involved in their youths’ care. All also favoured a community-based location that specializes in mental health care, with 24/7 e-health services offered in tandem with in-person care during flexible working hours.

Hawke and colleagues [20] methodology did not examine the IYS preferences of caregivers of justice-involved youth. This is an important question, given the high prevalence of MHS challenges and associated impairment among justice-involved youth and their families [1,22,23], yet lower rates of caregiver engagement [15,24,25].

## The Present Data Analysis

This data analysis was a secondary analysis of data gathered from a larger Ontario-wide DCE examining the integrated youth services (IYS) preferences of youth, caregivers, and service provider groups [19,20,21]. We extended this work by comparing the IYS preferences of caregivers of justice-involved youth and caregivers of non-justice-involved youth. Specifically, the following research question was explored: What components of IYS do caregivers of justice-involved youth deem to be the most important in meeting their youths’ MHS service needs, in comparison with caregivers of non-justice-involved youth?

## Method

### Survey Development

Survey development was based on guidelines on conjoint analysis experimental design [26]. In brief, phase one involved a review of characteristics of integrated models of care found in the literature [27], and phase two involved drafting attributes and levels to be included in the DCE. Drafts were edited through an iterative process involving the collaboration of research and clinical staff, youth, and caregivers. In the final phase of development, the DCE was piloted among four caregivers and four youth at two youth-serving mental health agencies within Ontario as part of the larger study conducted in July 2019.

### Participants

A total sample of 250 caregivers (*n* = 46 caregivers of justice-involved youth; *n* = 204 caregivers of non-justice-involved youth) participated in the current data analysis. To participate, caregivers had to self-identify as having lived experience caring for youth between the ages of 14 and 29 years with MHS challenges. Previous MHS service use was not required for inclusion, nor was caregiver’s personal involvement in the justice-system. A total of 346 caregivers were recruited from September 2019 to January 2020. Of this sample, 96 caregivers were excluded from the current data analysis because they did not meet eligibility criteria per above. Of the remaining 250 caregivers, *n* = 46 endorsed their youth having legal system involvement *and* either their youth as having legal charges *or* being arrested, *or* on probation and were therefore included in the analyses involving caregivers of justice-involved youth.

### Procedure

This data analysis was approved by The Centre for Addiction and Mental Health and Toronto Metropolitan University. Participants were recruited by circulating a survey link in email format, leveraging a database of over 600 MHS community agencies in Ontario [28]. Informed consent was obtained prior to survey completion via the survey link. The DCE and a questionnaire regarding sociodemographic information and service use was electronically delivered to participants using Sawtooth Software, Version 9 [29]. Median survey completion time was 23.67 minutes.

### Measures

#### Youth Justice Involvement

Justice involvement of youth was assessed via the sociodemographic questionnaire. Caregivers were asked “*Has your youth had legal system involvement?*” Respondent choices were: “*No, never*”; “*Yes, in the past 12 months*”; or “*Yes, more than a year ago*.” Caregivers who endorsed that their youth has ever had legal system involvement and endorsed at least one of the following met inclusionary criteria for caregivers of justice-involved youth; “*Does your youth have any legal charges?*”; “*Has your youth ever been arrested?*”; “*Is your youth currently on probation?*”

#### Discrete Choice Conjoint Experiment (DCE)

**Figure 1:**
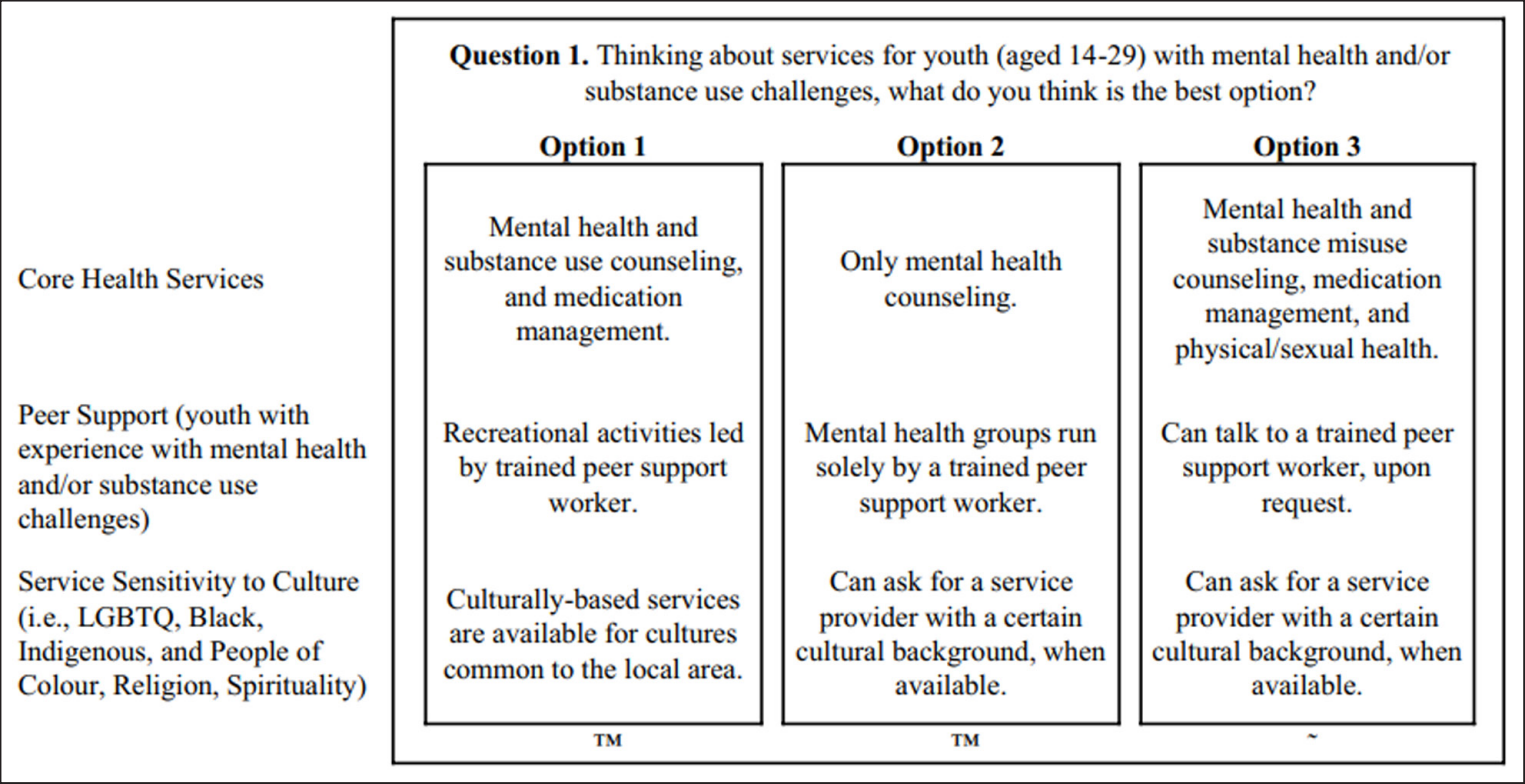
A sample DCE choice task.

The DCE contained a total of 12 attributes, each with four levels. Attributes included: (i) Core Health Services, (ii) Other Services, (iii) Caregiver Involvement, (iv) Peer Support, (v) Cultural Sensitivity, (vi) E-Health Services, (vii) Age Range, (viii) Time of Appointments, (ix) Wait Times, (x) Location, (xi) Engagement, and (xii) Information Sharing. Sawtooth Software, Version 9 [29] randomly produced 999 unique versions of the DCE and randomly assigned participants to complete one of the versions. Thirteen choice tasks were shown to each participant. Each choice task contained three concepts per task and three levels per concept and was administered using a Balanced Overlap Method (i.e., no duplicate concepts were permitted within the same task). One task was identical across all participants to test for respondent reliability. See Figure 1 for a sample DCE task.

### Data Analyses

Analyses were run in four stages using Sawtooth Software Hierarchical Bayes Estimation for Choice-Based Conjoint Data, Version 9 [29] and IBM SPSS Statistics, Version 27.0 [30].

#### Stage 1

A first-choice simulation was run to test the reliability of the DCE, with caregiver segment (caregivers of justice-involved youth vs. caregivers of non-justice-involved youth) entered as a covariate. How well the model predicted participant responses was determined by comparing caregiver responses on the fixed-choice task to predicted participant responses based on utility values generated by Hierarchical Bayes (HB) estimates.

#### Stage 2

An HB estimation was conducted to determine Attribute Importance Scores, with caregiver segment entered as a covariate. Importance scores were calculated as percentages, with all scores per participant group totaling 100% for the full set of 12 attributes. A MANOVA was performed, with caregiver segment as the independent variable and the Importance Scores for each attribute as the dependent variables to determine between-group differences in attribute preference. Where MANOVAs were significant, univariate ANOVAs with Bonferroni-corrected *p*-values were run to determine whether attribute preferences were statistically different between groups.

#### Stage 3

Standardized, zero-centered individual utility values generated by HB estimation were used to analyze group differences in preferences among levels within each attribute. A series of MANOVAs was conducted to determine which levels distinguish participant groups, with caregiver segment as the independent variable and utility values for each level within an attribute as the dependent variables. Where MANOVAs were statistically significant, univariate ANOVAs with Bonferroni corrections were computed to identify which preferred levels differed between participant groups.

#### Stage 4

A randomized first choice simulation was run to predict the percentage of caregivers of justice-involved and non-justice-involved youth who would prefer specified combinations (or *scenarios*) of IYS characteristics. Specifically, for each of the 12 attributes, the level most preferred by the caregivers of justice-involved youth was compared to the level most preferred by caregivers of non-justice-involved youth.

## Results

### Participant Characteristics

#### Sociodemographic information

Sociodemographic information with Chi-square statistics and effect sizes are displayed in Table 1. Caregivers of justice-involved youth (*M_age_* = 52.74, *SD* = 7.92) and non-justice-involved youth (*M_age_* = 51.18, *SD* = 8.03) were similar in age, *t*(237) = 1.16, *p* = .248, *d* = 0.20. Almost all caregivers of justice-involved youth (97.7%) and non-justice-involved youth (91.0%) identified as women, with the large majority being White (89.1% and 82.8%, respectively). A statistically significant between-group difference was found regarding employment, with more caregivers of justice-involved youth reporting being employed (80.0%) when compared to caregivers of non-justice-involved youth (64.5%).

**Table 1:** Sociodemographic and clinical characteristics of caregivers of justice-involved (C-YJ) and caregivers of non-justice-involved (C-non-YJ) participants, with significance tests and effect sizes.


GROUP	C-YJ	C-non-YJ	χ^2^ (*df*)	*p*	Φ

	(*n* = 46)	(*n* = 204)			

*Gender*	*n* (%)	*n* (%)	2.19 (2)	.335	.095^1^

Woman	42 (97.7)	183 (91.0)			

Man	1 (2.3)	17 (8.5)			

Transgender/non-binary	0 (0.0)	1 (0.5)			

*Race/Ethnicity*			4.12 (4)	.392	.128^1^

White	41 (89.1)	168 (82.8)			

Other	1 (2.2)	20 (9.9)			

Asian	3 (6.5)	10 (4.9)			

Black	0 (0.0)	3 (1.5)			

Indigenous	1 (2.2)	2 (1.0)			

*Socioeconomic Status*			2.01 (3)	.566	.090^1^

Live comfortably	27 (58.7)	125 (61.3)			

Meets needs with a little left	13 (28.3)	45 (22.1)			

Just meets basic expenses	6 (13.0)	28 (13.7)			

Does not meet basic expenses	0 (0.0)	6 (2.9)			

*Marital Status*			1.16 (2)	.560	.068

Married or common law	29 (65.9)	150 (73.5)			

Single, separated, or divorced	13 (29.5)	45 (22.1)			

Other	2 (4.5)	9 (4.4)			

*Region of Residence*			4.05 (2)	.132	.128

Rural to Small Population	11 (23.9)	25 (12.4)			

Medium Population	8 (17.4)	36 (17.9)			

Large Urban Population	27 (58.7)	140 (69.7)			

*Physical Health*			.998 (1)	.318	.063

Good to excellent	42 (93.1)	175 (85.8)			

Fair to poor	4 (8.7)	29 (14.2)			

*Mental Health*			.630 (1)	.466	.050

Good to excellent	36 (78.3)	148 (72.5)			

Fair to poor	10 (21.7)	56 (27.5)			

*Education Level*			.270 (2)	.874	.034

High school or less	2 (4.8)	10 (5.2)			

Some college/university	7 (16.7)	26 (13.6)			

Graduated college/university	33 (78.6)	155 (81.2)			

*Employed*			4.01 (1)	.045	.127

Yes	36 (80.0)	131 (64.5)			

No	9 (20.0)	72 (35.5)			


^1^ Cramer’s V (V∈) was used as a measure of effect size due to small cell sizes.

Sociodemographic characteristics of justice-involved and non-justice-involved youth, as reported by their caregivers, were also compared. Sociodemographic information, Chi-square statistics, and effect sizes are displayed in Table 2. Most caregivers of justice-involved and non-justice-involved youth reported being the parent of the youth they were reporting on (89.1% and 93.2%, respectively). On average, justice-involved youth (*M_age_* = 22.22, *SD* = 4.30) were approximately two years older than non-justice-involved youth (*M_age_* = 20.40, *SD* = 4.18). This difference reached statistical significance and represented a small to medium effect, *t*(248) = 2.65, *p* = .009, *d* = 0.43. A statistically significant difference in gender was also found between youth as reported by caregivers, with 69.6% of justice-involved youth being boys/young men and 28.3% being girls/young women; one justice-involved youth (2.2%) was identified as transgender or gender diverse. In contrast, 36.9% of non-justice-involved youth as reported by their caregivers identified as boys/young men, 52.7% as girls/young women, and 10.3% were identified as transgender or gender diverse. Furthermore, caregivers reported that significantly more justice-involved youth were not enrolled in school (67.4%) compared to non-justice-involved youth (40.4%). The remaining sociodemographic features of justice-involved and non-justice-involved youth, as reported by caregivers, were not statistically different.

**Table 2:** Sociodemographic and clinical characteristics of justice-involved (YJ) non-justice-involved (non-YJ) youth as reported by caregivers, with significance tests and effect sizes.


GROUP	YJ	NON-YJ	χ^2^ (*df*)	*p*	Φ

	(*n* = 46)	(*n* = 204)			

*Gender*	*n* (%)	*n* (%)	16.77 (2)	<.001	.260^1^

Woman	13 (28.3)*	107 (52.7)*	9.0 (1)	.003	

Man	32 (69.6)*	75 (36.9)*	16.0 (1)	<.001	

Transgender/non-binary	1 (2.2)	21 (10.3)	3.24 (1)	.072	

*Race/Ethnicity*			4.81 (4)	.308	.139^1^

White	36 (78.3)	159 (78.3)			

Other	5 (10.9)	32 (15.8)			

Asian	3 (6.5)	7 (3.4)			

Black	0 (0.0)	3 (1.5)			

Indigenous	2 (4.3)	2 (1.0)			

*Relationship to Youth*			1.90 (3)	.595	.087^1^

Parent	41 (89.1)	190 (93.1)			

Grandparent	0 (0.0)	2 (1.0)			

Sibling or other relative	2 (4.3)	5 (2.5)			

Other	3 (6.5)	7 (3.4)			

*Physical Health*			2.24 (1)	.135	.095

Good to excellent	24 (52.2)	130 (64.0)			

Poor to fair	22 (47.8)	73 (36.0)			

*Mental Health*			.851 (1)	.356	.058

Good to excellent	4 (8.7)	28 (13.7)			

Poor to fair	42 (91.3)	176 (86.3)			

*Student Status*			11.03 (1)	.001	.210

Part- or full-time enrollment	15 (32.6)	121 (59.6)			

Not enrolled	31 (67.4)	82 (40.4)			

*Education Level*			3.95 (2)	.139	.126

High school or less	37 (80.4)	134 (65.7)			

Some college/university	5 (10.9)	45 (22.1)			

Graduated college/university	4 (8.7)	25 (12.3)			

*Employment Status*			2.34 (3)	.506	.097^1^

Full-time	5 (10.9)	22 (10.8)			

Part-time	8 (17.4)	54 (26.6)			

Unemployed	29 (63.0)	105 (51.7)			

Other	4 (8.7)	23 (10.8)			

*Housing*			6.66 (4)	.070	.186^1^

Lives independently	3 (6.5)	14 (6.9)			

With a partner	5 (10.9)	11 (5.4)			

With family	29 (63.0)	151 (74.0)			

With friends	0 (0.0)	10 (4.9)			

Other/transitional-housing	9 (19.6)	18 (8.8)			


* Statistically significant at the Bonferroni corrected *p*-value of .017. ^1^ Cramer’s V (V∈) was used as a measure of effect size due to small cell sizes.

### Discrete Choice Conjoint Experiment

#### Stage 1

The “hit-rate” of the DCE was 88.0%, far greater than the chance rate of 33.3%, suggesting that the DCE model was reliable.

#### Stage 2

Table 3 illustrates the relative importance of each attribute by participant group. In order of most to least important, the top three most important attributes for caregivers of justice-involved youth were (1) Information Sharing, (2) Caregiver Involvement, and (3) Wait Time. For caregivers of non-justice-involved youth, the top three most important attributes were (1) Information Sharing, (3) Wait Time, and (3) Caregiver Involvement. The least important attributes for both groups were (1) Peer Support, (2) Cultural Sensitivity, and (3) Engagement.

**Table 3:** The relative importance of each IYS attribute for caregivers of justice-involved and non-justice-involved youth, and between-group MANOVA Pillai’s Trace statistic.


	JUSTICE-INVOLVED		NON- JUSTICE-INVOLVED				

ATTRIBUTE	*n* = 46		*n* = 204		*F* (*df*)	*p*	η*_p_^2^*
	
	*M*	*SD*	*M*	*SD*			

1. Core Health Services	8.01%	1.65	6.90%	2.16	10.74 (1)^a^	.001*	.042

2. Other Services	9.64%	1.75	8.27%	1.92	19.56 (1)	<.001*	.073

3. Caregiver Involvement	11.68%	2.70	11.15%	2.50	1.64 (1)	.201	.007

4. Peer Support	5.70%	1.72	6.20%	2.33	1.89 (1)^a^	.170	.008

5. Cultural Sensitivity	4.97%	2.16	5.76%	2.19	4.87 (1)	.028	.019

6. E-Health Services	9.17%	1.92	10.25%	1.98	11.29 (1)	<.001*	.044

7. Age Range	6.68%	1.87	7.45%	2.55	3.75 (1)^a^	.054	.015

8. Time of Appointments	8.47%	2.04	7.91%	2.49	2.00 (1)	.158	.008

9. Wait Time	9.86%	2.37	11.89%	2.50	25.20 (1)	<.001*	.092

10. Location	8.16%	1.78	7.91%	1.96	0.64 (1)	.425	.003

11. Engagement	3.77%	1.70	3.98%	1.68	0.53 (1)	.466	.002

12. Information Sharing	13.90%	2.61	12.35%	2.96	10.82 (1)	.001*	.042

Total	100%		100%				


IYS = Integrated Youth Service. ^a^ Levene’s test of equality of error variance statistically significant. * Statistically significant at the Bonferroni corrected *p*-value of .0042.

Pillai’s Trace test was significant, *V* = .213, *F*(11, 238) = 5.87, *p* < .001, η*_p_^2^* = .213, indicating statistically significant group differences in the relative importance of attributes. Univariate ANOVAs with Bonferroni corrected *p*-values demonstrate statistically significant between-group differences in the importance of the following attributes: Core Health Services, Other Services, and Information Sharing were significantly more important to caregivers of justice-involved youth when compared to caregivers of non-justice involved youth (small to medium effect). In contrast, E-Health Services (small to medium effect) and Wait Time (medium to large effect) were given higher importance among caregivers of non-justice-involved youth when compared to caregivers of justice-involved youth.

#### Stage 3

Although statistically significant effects were found within all 12 attributes, caregivers across both groups were unanimous in what their most preferred level among each attribute was, apart from family counselling. Caregivers of justice-involved youth showed a statistically significant greater preference for involvement in family counselling when compared to caregivers of non-justice-involved youth (medium to large effect). Results are presented in Table 4.

**Table 4:** Utility values of IYS levels for caregivers of justice-involved and non-justice-involved youth, and between-group MANOVA Pillai’s Trace statistic on attributes with follow-up Bonferroni corrected ANOVA results on levels.


	JUSTICE-INVOLVED		NON-JUSTICE-INVOLVED					

ATTRIBUTE LEVELS	*n* = 46		*n* = 204		*V*	*F*(*df*)	*p*	η*_P_^2^*
	
	*M*	*SD*	*M*	*SD*				

1. Core Health Services					.261	28.92 (3,246)	<.001	.261

Only mental health counseling.	–59.82	12.05	–43.56	16.00		42.11 (1)	<.001*	.145

Mental health and substance misuse counseling.	15.00	12.01	–2.22	13.50		63.49 (1)	<.001*	.204

Mental health and substance misuse counseling, and medication management.	14.87	10.59	11.33	12.30		3.27 (1)	.072	.013

Mental health and substance misuse counseling, medication management, and physical/sexual health.	29.95	16.50	34.45	20.88		1.87 (1)	.173	.007

2. Other Services					.091	8.19 (3,246)	<.001	.091

Education and employment services.	–5.65	19.27	–9.28	18.93		1.37 (1)	.243	.005

Housing, shelter and income support services.	16.57	17.41	18.11	20.68		.220 (1)	.640	.001

Legal support services.	–62.72	14.32	–52.42	17.28		14.17 (1)	<.001*	.054

Choice of education, employment, housing, income support, and legal support services.	51.80	11.96	43.58	12.16		17.27 (1)	<.001*	.065

3. Caregiver Involvement					.160	15.60 (3,246)	<.001	.160

No caregiver involvement.	–83.90	19.29	–84.22	20.67		.009 (1)	.925	.000

Caregivers receive own counseling.	–5.37	17.65	15.16	21.29		37.01 (1)	<.001*	.130

Caregivers involved in family counseling with youth, with youth consent.	51.23	21.14	34.51	21.29		23.21 (1)	<.001*	.086

Caregivers involved in decisions regarding youth counseling, with youth consent.	38.05	17.46	34.55	20.71		1.13 (1)	.288	.005

4. Peer Support					.138	13.07 (3,246)	<.001	.138

Recreational activities led by trained peer support worker.	–22.72	13.63	–28.93	16.36		5.73 (1)	.017	.023

Can talk to a trained peer support worker, upon request.	–9.66	12.03	2.57	15.50		25.19 (1)	<.001*	.092

Mental health groups run solely by a trained peer support worker.	–7.31	17.44	–11.49	21.69		1.49 (1)	.224	.006

Youth can be matched to an ongoing trained peer support worker to learn life skills and help them with services they need.	39.69	15.05	37.85	18.82		.384 (1)^a^	.536	.002

5. Cultural Sensitivity					.392	52.96 (3,246)	<.001	.392

Cultural background is not considered when picking a service or service provider.	–2.85	20.01	–27.36	21.04		51.81 (1)	<.001*	.173

Can ask for a service provider with a certain cultural background, when available.	–10.70	11.83	9.33	11.13		118.78 (1)	<.001*	.324

Services are culturally sensitive and trauma-informed.	30.39	22.08	26.14	26.85		.998 (1)	.319	.004

Culturally-based services are available for cultures common in the local area.	–16.84	14.58	–8.11	16.41		11.04 (1)	<.001*	.043

6. E-Health Services					.079	7.02 (3,246)	<.001	.079

No e-health or electronic services.	–41.09	18.33	–51.33	23.07		7.92 (1)	.005*	.031

Can schedule or reschedule appointments via email, text or online.	24.28	11.95	34.52	15.93		16.82 (1)^a^	<.001*	.064

E-health services are offered 24/7 alongside in-person services during office hours.	56.69	15.13	56.37	21.32		.009 (1)	.924	.000

All services are delivered only through a website, e-mail, text, or phone app.	–39.88	19.94	–39.56	22.40		.008 (1)	.929	.000

7. Age Range					.100	9.07 (3,246)	<.001	.100

Services for ages 12–24, in a youth-only setting.	–2.58	21.68	12.55	23.16		16.38 (1)	<.001*	.062

Services for ages 12–29, in a youth-only setting.	28.22	14.18	24.50	21.74		1.23 (1)^a^	.269	.005

Services for ages 12–24, in a setting that also has services for children 0–12.	–39.26	18.27	–43.54	22.92		1.40 (1)	.238	.006

Services for ages 12–29, in a setting that also has services for adults 29+.	13.62	27.42	6.49	33.49		1.81 (1)	.180	.007

8. Time of Appointments					.133	12.55 (3,246)	<.001	.133

Monday to Friday, 9AM-5PM.	–51.71	16.37	–44.82	21.84		4.06 (1)	.045	.016

Monday to Friday, 9AM-9PM.	–11.77	14.69	–12.69	19.10		.096 (1)^a^	.757	.000

Monday to Friday, 9AM-9PM, and Saturday, 9AM-5PM.	44.18	14.86	28.36	17.46		32.44 (1)	<.001*	.116

24/7.	19.29	25.40	29.15	32.07		3.81 (1)^a^	.052	.015

9. Wait Time					.155	15.08 (3,246)	<.001	.155

See a counselor for the first time immediately, during office hours.	63.09	17.39	72.18	20.37		7.86 (1)	.005*	.031

See a counselor for the first time after about 72 hours.	23.16	12.60	37.63	14.56		38.83 (1)	<.001*	.135

See a counselor for the first time after about 1 month.	–36.98	16.24	–46.70	17.85		11.49 (1)	.001*	.044

See a counselor for the first time after more than 1 month.	–49.27	20.23	–63.10	22.09		15.17 (1)	<.001*	.058

10. Location					.143	13.63 (3,246)	<.001	.143

Building or office in the community that specializes in mental health services.	53.16	14.61	39.42	18.54		22.14 (1)	<.001*	.082

Youth cafe and recreation centre.	1.66	21.00	21.52	25.40		24.35 (1)	<.001*	.089

Hospital or doctor’s office.	–11.50	15.62	–15.26	23.68		1.06 (1)^a^	.305	.004

School setting.	–43.32	13.75	–45.68	15.89		.865 (1)	.353	.003

11. Engagement					.252	27.60 (3,246)	<.001	.252

Youth and caregivers give feedback, e.g., anonymous surveys.	–2.72	15.04	–10.92	16.90		9.18 (1)	.003*	.036

Youth and caregivers are on staff at the organization.	–18.67	14.67	–0.49	14.38		59.61 (1)	<.001*	.194

Youth and caregivers are on an advisory group that gives feedback on services and evaluation.	13.79	15.33	19.00	16.27		3.92 (1)	.049	.016

Youth and caregivers play a leadership role in making decisions for the organization.	7.60	15.56	–7.59	16.77		31.61 (1)	<.001*	.113

12. Information Sharing					.105	9.62 (3,246)	<.001	.105

No sharing of personal information with caregivers.	–103.90	22.66	–89.96	26.54		10.89 (1)	.001*	.042

All personal information is available to caregivers, with youth consent.	31.10	26.75	27.19	31.00		.628 (1)	.429	.003

Service provider decides what information to share with caregivers, with youth consent.	12.48	20.43	15.04	24.43		.437 (1)	.509	.002

Youth and service provider work together to decide what personal information to share with caregivers and how that can be helpful.	60.32	13.52	47.73	17.89		20.14 (1)	<.001*	.075


^a^ Levene’s test of equality of error variance statistically significant. * Statistically significant at the Bonferroni corrected *p*-value of .0125.

##### Core Health Services

The most preferred combination of core health services among both groups was “Mental health and substance misuse counseling, medication management, and physical/sexual health,” with no statistically significant differences between groups. Both groups deemed “Only mental health counseling” as the least preferred core health service level, however, caregivers of justice-involved youth found it significantly less acceptable than caregivers of non-justice-involved youth (large effect).

##### Other Services

Both groups most preferred a “Choice of education, employment, housing, income support, and legal support services.” However, caregivers of justice-involved youth found this combination of services significantly preferable than caregivers of non-justice-involved youth (medium effect). Both groups least preferred “Legal support services” as the only other service offered. However, caregivers of justice-involved youth found this significantly less preferable (small to medium effect).

##### Caregiver Involvement

Caregivers of justice-involved youth most preferred the level “Caregivers involved in family counseling with youth, with youth consent,” while caregivers of non-justice-involved youth preferred this significantly less (large effect). Caregivers of non-justice-involved youth most preferred “Caregivers involved in decisions regarding youth counseling, with youth consent.” Caregiver of justice-involved youth also showed a preference for this level, with no statistically significant difference between groups. Both groups least preferred “No caregiver involvement,” with no statistically significant differences between groups.

##### Peer Support

Both groups exhibited the most preference for “Youth can be matched to an ongoing trained peer support worker to learn life skills and help them with services they need,” and the least preference for “Recreational activities led by trained peer support worker,” with no statistically significant differences between groups.

##### Cultural Sensitivity

Both groups most preferred that “Services are culturally sensitive and trauma-informed,” with no statistically significant differences between groups. Caregivers of justice-involved youth demonstrated the least preference for the level “Culturally-based services are available for cultures common in the local area,” while caregivers of non-justice-involved youth found this level significantly more acceptable. Caregivers of non-justice-involved youth demonstrated the least preference for the level “Cultural background is not considered when picking a service or service provider,” while caregivers of justice-involved youth found this level significantly more acceptable.

##### E-Health Services

Both groups most preferred that “E-health services are offered 24/7 alongside in-person services during office hours,” with no statistically significant differences between groups. Although both groups deemed “No e-health or electronic services” as least preferable, caregivers of non-justice-involved youth found this significantly less preferable (small effect).

##### Age Range

Both groups most preferred “Services for ages 12–29, in a youth-only setting,” and least preferred “Services for ages 12–24, in a setting that also has services for children 0–12,” with no statistically significant differences between groups.

##### Time of Appointments

Both groups most preferred that appointments be offered “Monday to Friday, 9AM-9PM, and Saturday, 9AM-5PM,” however, caregivers of justice-involved youth preferred this significantly more than caregivers of non-justice-involved youth (large effect). Both groups least preferred that appointments be offered “Monday to Friday, 9AM-5PM,” with no statistically significant differences between groups.

##### Wait Time

The level “See a counselor for the first time immediately, during office hours,” had the highest utility value among all 48 levels within both groups, indicating the highest relative preference. Furthermore, caregivers of non-justice-involved youth preferred this level significantly more than caregivers of justice-involved youth (small effect). Both groups least preferred “See a counselor for the first time after more than1 month.” However, caregivers of non-justice-involved youth found this wait time significantly less preferable than caregivers of justice-involved youth (medium effect).

##### Location

Both groups most preferred that the location of services be offered in a “Building or office in the community that specializes in mental health services,” however, caregivers of justice involved-youth preferred this significantly more than caregivers of non-justice-involved youth (large effect). Both groups deemed a “School setting” to be the least preferred location, with no statistically significant differences between groups.

##### Engagement

Both groups most preferred that “Youth and caregivers are on an advisory group that gives feedback on services and evaluation,” with no statistically significant differences between groups. Rather, caregivers of non-justice-involved youth preferred that “Youth and caregivers are on staff at the organization” second-best. This significantly differed from caregivers of justice-involved youth, who preferred this level of engagement least (large effect). In contrast, caregivers of non-justice-involved youth least preferred that “Youth and caregivers give feedback, e.g., anonymous surveys,” while caregivers of justice-involved youth found this level of engagement significantly more acceptable (small effect).

##### Information Sharing

Both groups most preferred that the “Youth and service provider work together to decide what personal information to share with caregivers and how that can be helpful,” however, caregivers of justice-involved youth preferred this significantly more than caregivers of non-justice-involved youth (medium to large effect). Both groups least preferred “No sharing of personal information with caregivers.” Of note, this level was the least preferred among all 48 levels among both groups, however, caregivers of justice-involved youth found it significantly less preferable than caregivers of non-justice-involved youth (small to medium effect).

#### Stage 4

When caregivers of non-justice-involved youth’s top preferred level within each of the 12 attributes is included in a hypothetical IYS scenario (i.e., *Scenario 1*), 51.6% of caregivers of non-justice-involved youth would prefer the model, compared to only 25.1% of caregivers of justice-involved youth. When caregivers of justice-involved youth’s top preferred level within each of the 12 attributes is included in a hypothetical IYS scenario (i.e., *Scenario 2*), 48.4% of caregivers of non-justice-involved youth would prefer the model, slightly decreasing acceptability among this caregiver group. However, acceptability among caregivers of justice-involved youth increases substantially, to 74.9% (see Table 5).

**Table 5:** Randomized first choice simulation results of each caregiver group’s ideal IYS scenario, with standard errors and 95% confidence intervals.


	CAREGIVERS OF JUSTICE-INVOLVED YOUTH *n* = 46			CAREGIVERS OF NON-JUSTICE-INVOLVED YOUTH *n* = 204		
	
	SHARES OF PREFERENCE	*SE*	95% *CI*	SHARES OF PREFERENCE	*SE*	95% *CI*

*Scenario 1*	25.1%	4.5%	16.2%–33.9%	51.6%	2.7%	46.4%–56.9%

*Scenario 2*	74.9%	4.5%	66.1%–83.8%	48.4%	2.7%	43.1%–53.6%


*SE* = Standard Error. *CI* = 95% Confidence Interval. *Scenario 1* refers to when the most preferred level of caregivers of non-justice-involved is implemented across all 12 attributes. *Scenario 2* refers to when the most preferred level of caregivers of justice-involved is implemented across all 12 attributes.

## Discussion

This data analysis used a DCE to contrast the specific IYS component preferences of caregivers of justice-involved youth from caregivers of non-justice-involved youth to understand the IYS characteristics caregivers of justice-involved youth deem to be the most important in meeting their youths’ MHS service needs. Caregivers of justice-involved youth showed a unique preference for involvement in family counseling with their youth. This data analysis also demonstrates that, overall, caregivers of justice-involved and non-justice-involved youth were otherwise concordant in what they deem to be the most important service characteristics in forming integrated youth services. For example, caregivers in both groups deemed immediate access to service as their most preferred service characteristic, consistent with previous research exploring caregiver MHS service preferences [20,31,32]. Caregivers in both groups also prioritized their involvement in their youth’s services. It is notable that both caregiver groups showed a markedly low preference for “no sharing of personal information with caregivers.” This parallels findings from the Hawke and colleagues’ study [20], from which this analysis used the same data, which demonstrated that caregivers across latent classes prioritized information sharing and negatively endorsed no information sharing. It is also consistent with other literature demonstrating that for many caregivers of youth in Canada, involvement in their youths’ MHS is highly prioritized [33].

In addition to the above, both groups preferred the opportunity for their youth to engage in a range of core health services that include mental health and substance misuse counseling, medication management, and physical/sexual health. This is consistent with findings from the Hawke and colleagues [20] study, as well as other international literature exploring the service preferences of caregivers [34]. Moreover, preference for a broad range of additional IYS services, namely, education, employment, housing, income support, and legal support services was congruent across both caregiver groups. Notably, both caregiver groups preferred a broad range of additional services (e.g., education and employment) even more strongly than a broad range of core health services, with the preference for these services especially strong among caregivers of justice-involved youth. This reflects what has been expressed repeatedly in the literature: Compared to non-justice-involved youth, justice-involved youth have pronounced challenges fulfilling their roles in school, work, and in the community [22,23]. Caregivers of justice-involved youth seem to recognize that the incorporation of these services are integral in treating their youth within an IYS framework, thus making them a strong preference, as demonstrated in the current data analysis.

Both caregiver groups agree that e-health services should be offered 24/7 alongside in-person services, consistent with previous research [20]. Notably, data for this analysis were collected prior to the COVID-19 pandemic, which was associated with stringent lockdown procedures in Canada, negatively impacting youth mental health as well as access to mental healthcare [35]. Therefore, it is plausible that preference for this service feature has become increasing important, to address both the growing need for youth MHS services and difficulty accessing in-person care during the pandemic [35].

Finally, both caregiver groups preferred that the location of services be offered in a community setting that specializes in mental health services. This is consistent with Hawke and colleagues [20] findings that caregivers across latent classes prefer community-based care and with other IYS models that use specialized community-based settings to increase accessibility to MHS care [8].

An important distinction among caregivers of justice-involved youth was their preference for family counselling. A simulation revealed that the inclusion of family counselling could potentially increase engagement among caregivers of justice-involved youth 3-fold. The prioritization of family counselling among caregivers of justice-involved youth is somewhat surprising, as previous research has demonstrated that caregivers of justice-involved youth may be less willing or able to engage in treatment with their youth than caregivers of non-justice-involved youth [7,14,15,24,25,36]. For example, justice-involved youth are more likely to be from single-parent households [24,25], which may impact caregivers’ available resources to play an active a role in the MHS treatment of their youth [24,25]. It is worth noting that justice-involved youth may desire less involvement than their caregivers, particularly during adolescence and early adulthood, a period in which youth increasingly desire autonomy [7]. Higher levels of conflict among the families of justice-involved youth may also impact the preference for caregiver involvement [14,25]. However, findings from this data analysis demonstrated that the preference for family counselling for caregivers of justice-involved youth was not only significantly greater than caregivers of non-justice-involved youth, but was among their most preferred service characteristics. Caregiver involvement in counselling is paramount to a variety of evidence-based treatments for youth involved in the criminal justice system [9,10,11]. The alignment between caregivers of justice-involved youth preference for family counselling with best practices should therefore be capitalized on in integrated youth services.

Unfortunately, three of four simulations conducted in this data analysis demonstrated that full or partial IYS models would only engage about half of all caregivers across justice-involved and non-justice-involved groups. While this analysis did find one model that is predicted to engage approximately 75% of caregivers of justice-involved youth, the same model is predicted to engage less than half of caregivers of justice-involved youth. The overall low predicted engagement of caregivers across both groups in most simulations may reflect latent classes within our caregiver sample [20,37]. For example, although Hawke and colleagues [20] found that caregivers had similar preferences in a variety of domains, latent class models demonstrated that three caregiver latent classes differed in their prioritization of comprehensive and integrated services, service access, and caregiver involvement. Therefore, to increase caregiver engagement, a broad range of flexible service options may be required in designing IYS models. Findings from this data analysis suggest that flexible service options in the domains of caregiver involvement should be provided, especially when aiming to engage caregivers of justice-involved youth.

### Limitations

Caregivers of justice-involved youth participants were predominantly White. The majority were also highly educated, affluent women in stable partnerships, with sound physical and mental health. The sociodemographic features of the present sample were not characteristic of the population of caregivers of justice-involved youth in Canada [38]. This may account for findings that culturally sensitive services were not prioritized among caregivers. Due to the underrepresentation of BIPOC caregivers in this data analysis, conclusions regarding the desire for culturally sensitive services should not be made. It is suspected that culturally sensitive services would have been prioritized if the study sample were more ethnically diverse. Notably, it is possible that the larger study experienced a poor response rate among caregivers of justice-involved youth, especially BIPOC caregivers, due to barriers related to confidentiality and trust in MHS care [14,15] and lack of access to culturally sensitive services among this population [24,25]. If caregivers of justice-involved youth are not engaging in agencies that serve youth with MHS needs, recruitment efforts would not have reached them. Replication of this study with participants who better reflect the composition of caregivers of justice-involved youth is needed.

Data for the larger study were collected prior to the COVID-19 pandemic. In addition to the pandemic’s impact on youth mental health and substance use [35], the pandemic has also impacted how MHS services are delivered to youth [39,40]. Such changes may impact the post-pandemic MHS service preferences of caregivers. As such, a replication of this data analysis in post-pandemic times is warranted.

### Conclusions

This was the first known data analysis to examine and contrast the specific IYS component preferences of caregivers of justice-involved youth compared to their non-justice-involved counterparts. Both caregiver groups demonstrated preferences for collaboration in their youth’s care, and fast access to a variety of services that encompasses education, employment, housing, income support, and legal support services. Both groups also exhibited a preference for a variety of core health services offered in a community setting, with the incorporation of e-health services. These service characteristics should form the foundation of integrated youth services.

In addition to these above foundational elements, involvement in family counselling was preferred among caregivers of justice-involved youth. An IYS model that is designed to include the above foundational elements in addition to flexible options for caregiver involvement in counselling has the potential to enhance service utilization in caregivers of justice-involved youth, ultimately leading to better outcomes for their families, their youth, and their communities.
